# Nasopharyngeal metagenomic deep sequencing data, Lancaster, UK, 2014–2015

**DOI:** 10.1038/sdata.2017.161

**Published:** 2017-10-24

**Authors:** Kate V. Atkinson, Lisa A. Bishop, Glenn Rhodes, Nicolas Salez, Neil R. McEwan, Matthew J. Hegarty, Julie Robey, Nicola Harding, Simon Wetherell, Robert M. Lauder, Roger W. Pickup, Mark Wilkinson, Derek Gatherer

**Affiliations:** 1 Division of Biomedical & Life Sciences, Faculty of Health & Medicine, Lancaster University, Lancaster LA1 4YT, UK; 2 Royal Lancaster Infirmary, Ashton Road, Lancaster LA1 4RP, UK; 3 Centre for Ecology & Hydrology, Lake Ecosystems Group, Lancaster Environment Centre, Lancaster University, Lancaster LA1 4AP, UK; 4 UMR_D 190, Emergence des Pathologies Virales, Aix-Marseille University, 27 Bd Jean Moulin, Marseille cedex 05 13005, France; 5 Institute of Biological, Environmental & Rural Sciences, Aberystwyth University, Aberystwyth SY23 3DA, UK; 6 Queen Square Medical Practice, 2 Queen Square, Lancaster LA1 1RP, UK

**Keywords:** Clinical microbiology, Infectious diseases, RNA sequencing, Metagenomics, Virology

## Abstract

Nasopharyngeal swabs were taken from volunteers attending a general medical practice and a general hospital in Lancaster, UK, and at Lancaster University, in the winter of 2014–2015. 51 swabs were selected based on high RNA yield and allocated to deep sequencing pools as follows: patients with chronic obstructive pulmonary disease; asthmatics; adults with no respiratory symptoms; adults with feverish respiratory symptoms; adults with respiratory symptoms and presence of antibodies against influenza C; paediatric patients with respiratory symptoms (2 pools); adults with influenza C infection (2 pools), giving a total of 9 pools. Illumina sequencing was performed, with data yields per pool in the range of 345.6 megabases to 14 gigabases after removal of reads aligning to the human genome. The data were deposited in the Sequence Read Archive at NCBI, and constitute a resource for study of the viral, bacterial and fungal metagenome of the human nasopharynx in healthy and diseased states and comparison with other metagenomic studies on the human respiratory tract.

## Background & Summary

Respiratory infectious disease is a major cause of morbidity and mortality in the elderly, infants, the immune-compromised and those with underlying serious pulmonary conditions such as asthma or cystic fibrosis. It is also a source of considerable economic damage through working days lost to illness in adults (ref. 1). Fungi, viruses and bacteria can all cause respiratory infections, but aside from a handful of high profile pathogens such as influenza A virus, *Pneumocystis carinii*, respiratory syncytial virus and *Streptococcus*, the problem of respiratory infectious disease diagnosis, treatment and prevention remains neglected.

The sheer diversity of microbes causing respiratory infections that are indistinguishable on clinical examination, means that the vast majority of patients receive only a generic diagnosis of coryza or pharyngitis. Active identification of a causative agent is usually only performed in the event of the illness progressing to severe bronchitis or pneumonia. Despite the highly selective nature of diagnostic results obtained in this way, they offer many important insights. For instance, the WHO tracks the progress of influenza across the world (ref. 2) using percentages of samples testing positive for influenza viruses A/H1N1, A/H3N2 and B. Cumulatively, such diagnostic samples may demonstrate trends in respiratory illness over several years. Nickbakhsh, *et al.* (ref. 3) tested for 11 virus groups in >60,000 samples collected from >40,000 patients in the west of Scotland from 2005 to 2013, revealing extensive co-infections and a general increase in the respiratory viral load in the population, especially in infants, which remains unexplained.

Deep sequencing, which produces a metagenome reflecting the total microbial genetic content in a sample, offers an alternative to such highly targeted approaches, but is only just beginning to enter the routine diagnostic pipeline (ref. 4–6). Nevertheless, accumulation of human respiratory tract metagenomes from research projects is well underway, providing a resource for the generation of a comprehensive profile of the respiratory microbiome in both health and disease. Within the Sequence Read Archive (SRA), the tag ‘human nasopharyngeal metagenome’ identifies 41 BioProject entries as of 30th August 2017, including that reported here. Within this group of datasets, there are upper respiratory tract metagenomes associated with influenza, respiratory syncytial virus, allergy, intubation, asthma, macular degeneration, oesophageal reflux, rhinosinusitis and Parkinsonism as well as samples taken from healthy volunteers. Our project (ref. 7) initially focussed on the prevalence and severity of influenza C infection in Lancaster, UK, during the winter of 2014–2015. We performed Ilumina deep sequencing on 9 patient pools (Data Citations 1–9), as detailed in Table 1. Two of our 9 sequencing pools (Data Citations 8, 9) were from individuals diagnosed with influenza C infection via quantitative RT-PCR, from one of which we could partially assemble (ref. 7) the genome of a novel strain of influenza C, designated C/Lancaster/1/2015 (Data Citations 10–12). Within other sequencing pools we also detected the complete genomes of novel strains of human rhinovirus A22 (ref. 8), human papillomavirus 23 (ref. 9) and human papillomavirus 20 (ref. 10) (Data Citations 13–15).

**Figure 1:**
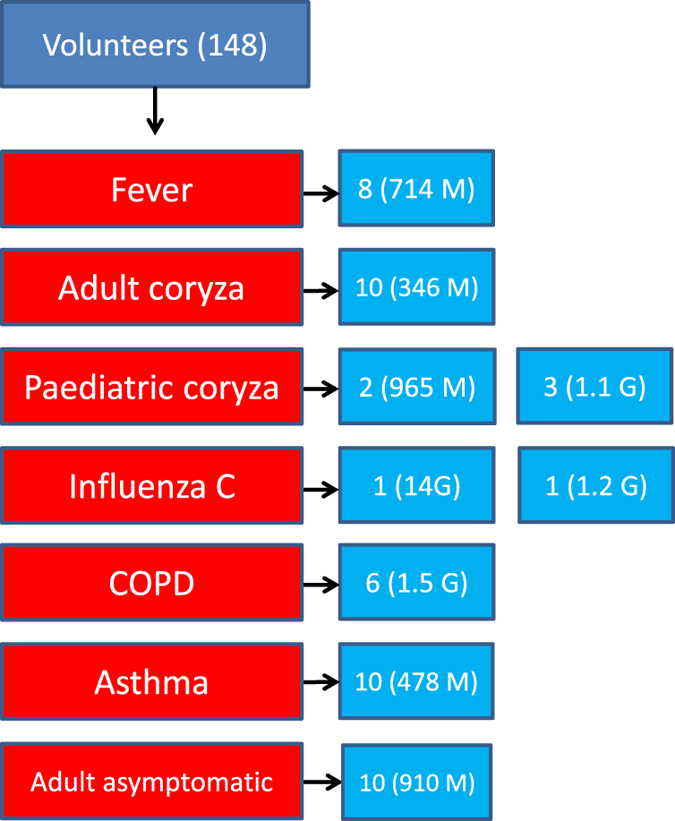
Clinical flowchart. From 148 nasopharyngeal swabs, 51 were chosen for allocation to 7 symptom groups, of which 2 were divided into two separate runs, making a total of 9 deep sequencing pools.

**Figure 2:**
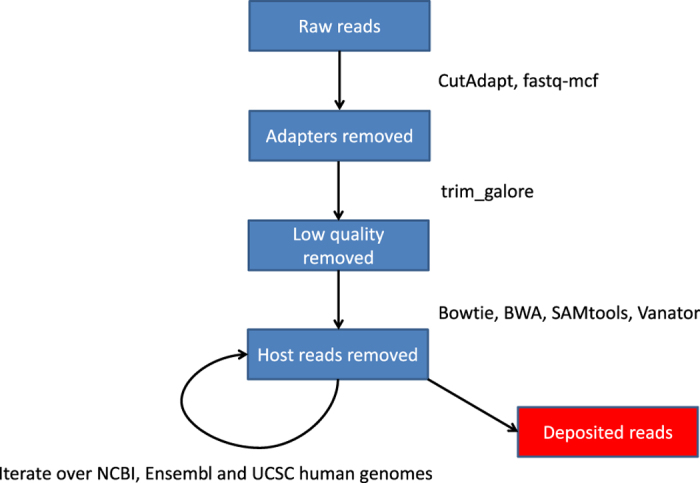
Read processing flowchart. The raw reads were cleaned and then subjected to sequential alignments to 3 versions of the human genome, with mapped reads discarded at each stage. The software used at each stage is shown.

Figure 1 summarises our clinical workflow. From the 148 samples taken, 51 were prioritised for allocation to the 9 sequence pools, based on high yield of RNA and/or clinical phenotype. Our interest in influenza C is reflected in the decision to devote 2 of our 9 sequencing pools to single individuals positive for influenza C by reverse transcription polymerase chain reaction (RT-PCR), one of whom was extensively sequenced (14 Gb). Paediatric coryza was similarly also prioritised (ref. 11) with two pools of 2 and 3 individuals respectively (Data Citations 1, 2). Figure 2 summarises how deep sequencing reads were processed. The raw deep sequencing output was first trimmed of adapter and other extraneous sequences, then groomed to remove reads of low quality score. To avoid the potential ethical complication of sequence from the genomes of the volunteers being present in the sample, three versions of the human genome were used to screen out reads of human origin. It should be noted, however, that we have not attempted to remove any of the known common bacterial contaminants of deep sequencing reactions (ref. 12).

Since we performed deep sequencing on extracted RNA (see Methods), reads are expected to be greatly biased towards expressed regions. Therefore, although we have previously used the data to derive 3 complete short viral genomes (ref. 8–10), detectable genomes of cellular microbes are more likely to be fragmentary. Nevertheless, the dataset may be used for the discovery of further novel viruses, or for comparative studies of the whole virome or bacteriome between the 7 different clinical states defined in the 9 samples. Some of the samples may be compared with other microbiome depositions in the SRA, for instance with BioProject PRJNA310124 (Data Citation 16) on asthma in children (ref. 13). The smaller sequence read sets, for instance SRX2310765 (Data Citation 3) which contains 346 Mb of sequence, are suitable for use as training data for the bioinformatics tools used in metagenomics.

## Methods

### Ethics

Ethical approval was granted by the UK National Research Ethics Service (NRES), reference 14/LO/1634, Integrated Research Application System (IRAS) Project 147631. The project was registered with the National Institute of Health Research (NIHR), UK, as part of the NIHR Clinical Research Network (UKCRN) Portfolio, ID 17799. All methods were carried out in accordance with the relevant guidelines and regulations. Informed consent was obtained from all volunteers of 18 years and older. For those under 18 years, informed parental consent was obtained, together with supervised assent of the volunteer.

### Patient recruitment & sample processing

Participants were approached in 3 locations in Lancaster, UK, (54.05°N 2.80°W) from November 2014 to May 2015: 1) Lancaster University, 2) a general medical practice, 3) hospital clinics. After informed consent was given, patients were allocated to a clinical category (Fig. 1). Nasopharyngeal swabs (MW951SENT, Medical Wire) were brushed over the rear wall of the nasopharynx of patients, and the tips then snapped off directly into Sigma Virocult medium.

RNA was extracted from the nasopharyngeal swabs using a MagMAX Viral RNA Isolation Kit (Ambion). cDNA was prepared using a High-Capacity RNA-to-cDNA Kit (Applied Biosystems, Life Technologies) and a Veriti Thermal Cycler (Applied Biosystems, Life Technologies). The samples were incubated at 37 °C for 60 min, before stopping the reaction at 95 °C for 5 min and then holding at 4 °C. Storage of completed reactions was at −20 °C. After selection of a subset of samples (Fig. 1), deep sequencing was performed using an Illumina Nextera XT library kit in 2×126 bp format on the Illumina HiSeq2500 system.

### Processing of raw deep sequencing data for quality control and to remove reads of human genome origin

Quality of Illumina deep sequencing reads was assessed using FastQC (see Technical Validation). Reads were then trimmed of adapters and other non-genomic elements using CutAdapt 1.1 (ref. 14) (https://pypi.python.org/pypi/cutadapt), fastq-mcf 0.11.3 (ref. 15) (https://expressionanalysis.github.io/ea-utils), and trim_galore (http://www.bioinformatics.babraham.ac.uk/projects/trim_galore/), within the Read_cleaner pipeline (Gatherer, unpublished, see Code Availability). Reads of less than 25 bases after trimming were discarded.

Read_cleaner implements the following commands:

- fastx_clipper -a ADAPTOR_SEQ -l 25 -n -v -M 5 -i INPUT_FILE -o TEMP_FILE
- trim_galore --length 25 --adapter ADAPTOR_SEQ --stringency 5 TEMP_FILE
- fastq-mcf adapters.fa TEMP_FILE -l 25 -o OUTPUT_FILE

cycling fastx_clipper and and trim_galore (which itself pipelines CutAdapt) over a set of common adaptor sequences, passing the output of each iteration to a temporary file, followed by application of fastq-mcf, which takes all the adapters in a single file. Minimum read length is specified as 25 for each of the 3 commands, but this is an operator-entered variable in Read_cleaner.

Ethical approval required that no genetic material remain within the samples which could enable identification of patients. Therefore, human genome and transcriptome sequences were removed by iterative alignment onto the NCBI, Ensembl and UCSC human iGenomes (http://support.illumina.com/sequencing/sequencing_software/igenome.html), first using bowtie 1.1.1 (ref. 16) (http://bowtie-bio.sourceforge.net/index.shtml), then BWA 0.7.12-r1039 (ref. 17) (http://bio-bwa.sourceforge.net). Following each alignment, extraction of unaligned reads was achieved using samtools 0.1.19 (ref. 18) (http://samtools.sourceforge.net) and the next alignment commenced. Bowtie, BWA and samtools were co-ordinated using the Vanator pipeline (ref. 19).

Vanator implements the following commands for bowtie and BWA:

- bwa aln GENOME READS>SAI_INDEX
- bwa samse GENOME SAI_INDEX READS>SAM
- bowtie -S -p 8 GENOME READS SAM
- samtools view -S SAM -b -o BAM
- samtools sort BAM BAM_FILE
- samtools index BAM
- bam2fastq --no-aligned --force --strict -o READS BAM

cycling over the three reference human genomes, each time retrieving the unaligned reads using bam2fastq.

Quality improvements were checked, again using FastQC (see Technical Validation).

### Identification of potential bacterial contaminants in the sequence pools

Potential contaminating genomes (produced by the presence of non-metagenome DNA contaminants in sequencing reagents) were hypothesised by reference to Salter *et al.* (ref. 12), and a representative screening set was selected as follows: NC010725 Methylobacterium populi BJ001; NC009485 Bradyrhizobium sp. BTAi1; NC014323 Herbaspirillum seropedicae SmR1; NC022438 Leifsonia xyli subsp. cynodontis DSM 46306; NC015675 Mesorhizobium opportunistum WSM2075; NC007794 Novosphingobium aromaticivorans DSM 12444; NC008313 Ralstonia eutropha H16 chromosome 1; NZCP009571 Sphingomonas taxi strain ATCC 55669; NZCP010409 Xanthomonas sacchari strain R1.

An assessment of the relative contribution of such contaminants was produced by aligning each sequence pool onto the genomes listed above using bowtie 1.1.1 (ref. 16) (http://bowtie-bio.sourceforge.net/index.shtml). However, no further action was taken to remove reads corresponding to these contaminants, or related species, and they remain in the data (see Technical Validation).

### Code availability

The unpublished Read_cleaner pipeline is freely available under CC-BY license (Data Citation 17) along with instructions for its use. Vanator (ref. 19) is available at https://sourceforge.net/projects/vanator-cvr.

## Data Records

The primary data discussed in this paper is the Illumina deep sequencing output available in Data Citations 1–9. The data format of the deep sequencing output is FASTQ (ref. 20), which adds sequencing quality data to the well-established FASTA nucleic acid sequence format (ref. 21). Viral genome sequences derived from the deep sequencing output by assembly (see Methods) are given in Data Citations 10–15. These are in GenBank format.

Table 1 cross-references the SRA BioSample, Experiment, Accession and Run references against the clinical sources and other data on each sequencing pool. These different accessions provide alternative access points to the data within SRA. They are intended to operate in a hierarchical manner, allowing for instance several sequencing ‘Runs’ to be derived from each ‘Experiment’, and several ‘Experiments’ to be performed on a single ‘BioSample’, and so on. In our work, the hierarchy is sparse, in that each ‘Sample’ was treated as a separate ‘Experiment’, and only one deep sequencing ‘Run’ performed on each.

Table 2 gives the proportion of reads aligning to the top two contaminants of each sequencing pool. In all pools, *Methylobacterium populi* is the most common contaminant, with more than 3% of reads in all pools except F, and up to 21% of reads in pool A, and the second most common is *Ralstonia eutropha*, with under 0.7% of reads in all pools. Other contaminants total 0.1 to 1.2% of reads.

## Technical Validation

### Deep sequencing read technical quality

FastQC (https://www.bioinformatics.babraham.ac.uk/projects/fastqc/) was used to check the quality of the Illumina sequencing reads, both in their raw state and after the cleaning processes described above in Methods. FastQC output is deposited in Data Citation 18.

### Deep sequencing reagent contaminants

Alignments of each sequencing pool to a set of representative common reagent contaminant genomes (ref. 12) was performed using bowtie 1.1.1 (ref. 16) (http://bowtie-bio.sourceforge.net/index.shtml). The BAM files, their BAI indexes and the contaminant reference genomes are deposited in Data Citation 19.

## Additional information

**How to cite this article:** Atkinson, K. V. *et al.* Nasopharyngeal metagenomic deep sequencing data, Lancaster, UK, 2014–2015. *Sci. Data* 4:170161 doi: 10.1038/sdata.2017.161 (2017).

**Publisher’s note:** Springer Nature remains neutral with regard to jurisdictional claims in published maps and institutional affiliations.

## Supplementary Material



## Figures and Tables

**Table 1:** Deep sequencing pools A-I, their sources and references.

**Set**	**BioSample**	**Source**	**Clinical**	**pool size**	**Experiment**	**SRA**	**Run**	**Bases**
A	SAMN05954283	Paediatric, Low RNA	Coryza	3	SRX2310763	SRS1768710	SRR4733499	1.1 G
B	SAMN05954284	Paediatric, High RNA	Coryza	2	SRX2310764	SRS1768711	SRR4733500	964.8 M
C	SAMN05954285	Adult, High fluC IgG	Coryza	10	SRX2310765	SRS1768712	SRR4733501	345.6 M
D	SAMN05954286	Adult	Fever	8	SRX2310766	SRS1768713	SRR4733502	714.3 M
E	SAMN05954287	Adult	Asymptomatic	10	SRX2310759	SRS1768706	SRR4733495	909.7 M
F	SAMN05954288	Adult	Asthmatic	10	SRX2310760	SRS1768707	SRR4733496	477.5 M
G	SAMN05954289	Adult	COPD	6	SRX2310761	SRS1768708	SRR4733497	1.5 G
H	SAMN05954290	adult	Influenza C positive	1	SRX2310762	SRS1768709	SRR4733498	14 G
I	SAMN05954291	Adult	Influenza C positive	1	SRX2310758	SRS1768705	SRR4733494	1.2 G
The number of bases per sequencing pool is given in gigabases (G) or megabases (M) for those pools generating less than 1G after removal of reads of human genome origin. COPD: chronic obstructive pulmonary disease. IgG: immunoglobulin G.								

**Table 2:** Proportion of common reagent contaminants per sequencing pool.

	**NC010725 M.populi**	**NC008313 R. eutropha**	**Others**
**Set**	**Proportion (%)**	**Proportion (%)**	**Proportion (%)**
A	21	0.3	0.6
B	14	0.2	0.5
C	3.0	0.1	0.1
D	20	0.4	0.7
E	12	0.6	1.2
F	0.9	0.1	0.2
G	9.1	0.7	1.0
H	16	0.2	0.5
I	12	0.2	0.5
The pools are labelled A-I as in Table 1. Proportions are calculated as number of reads aligning to a representative set of typical contaminants^12^, per total number of reads in that pool. M.populi: Methylobacterium populi; R.eutropha: Ralstonia eutropha. See Data Citation 19 for further details.			
